# An innovative treatment approach using iliac artery embolization and implanted port for chemotherapy in a patient with gluteal muscle metastasis of advanced colon cancer: a case report and literature review

**DOI:** 10.3389/fonc.2025.1630104

**Published:** 2025-09-02

**Authors:** Sen Wang, Heng Zong, Lei Tang, Yuan-dong Wei

**Affiliations:** Department of Medical Oncology, Anhui No.2 Provincial People’s Hospital, Hefei, China

**Keywords:** case report, colorectal cancer, iliac artery embolization, intra-arterial chemotherapy, gluteal muscle metastasis

## Abstract

Colorectal cancer (CRC) is a leading cause of cancer-related morbidity and mortality worldwide, with metastasis often resulting in significant treatment challenges. This case report describes a 54-year-old male patient who presented with a rectal cancer metastatic mass accompanied by erosion in the left gluteal area, diagnosed as poorly differentiated adenocarcinoma, along with significant cachexia and pelvic and lymph node metastases. Despite undergoing CAPEOX chemotherapy, no notable tumor shrinkage was observed. In light of this, a multidisciplinary consultation led to the adoption of an innovative approach that included transiliac artery chemotherapy embolization and the implantation of an arterial chemotherapy pump for mFOLFOX6 combined with bevacizumab. Following four cycles of reduced-dose arterial infusion chemotherapy, the tumor in the gluteal region demonstrated a remarkable reduction of approximately 90%. This case showcases a promising treatment modality that integrates localized arterial embolization and chemotherapy, along with supportive care. The approach significantly improved the patient’s quality of life and extended survival in individuals facing advanced colorectal cancer with gluteal muscle metastasis, suggesting that this therapy merits further investigation and potential implementation in clinical practice.

## Introduction

Colorectal cancer is one of the leading causes of cancer-related deaths worldwide (1, 2). In recent years, the continuous innovation and widespread application of diagnosis and screening techniques have significantly improved the quality of life and prognosis of patients. However, nearly 25% of all patients present with distant metastases at diagnosis (3). Colorectal cancer metastasizes with some degree of preference, commonly to the peritoneum, liver, lungs, and bones (4). There are few reports of skeletal muscle metastasis in colorectal cancer, especially involving the gluteal muscles (5–7). This study aims to document this rare pattern of metastasis and report an interventional treatment approach that combines iliac artery infusion chemotherapy, with the results potentially informing future therapeutic strategies.

## Case presentation

**Figure 1:**
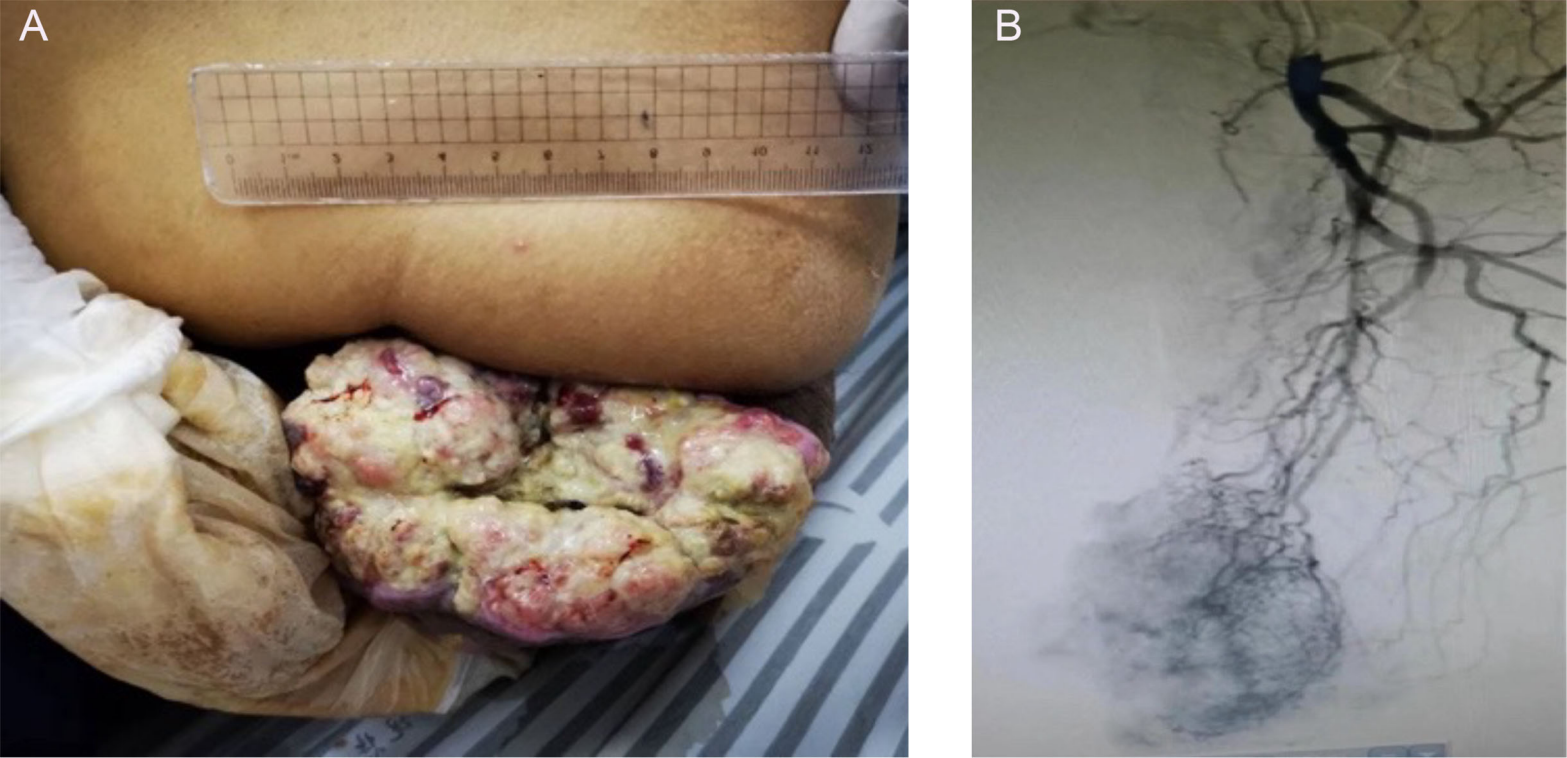
A large ulcerated mass on the left buttock at initial diagnosis **(A)** and angiographic vascular imaging of the metastatic tumor **(B)**.

A 54-year-old male patient was admitted to Anhui Provincial Second People’s Hospital in early October 2023, presenting with a large left gluteal mass that had been evident for approximately six months, accompanied by recently developed ulceration. PET-CT revealed increased fluorodeoxyglucose (FDG) uptake in a mass in the rectum, multiple nodular lesions in the pelvic and bilateral groin regions, and a mass in the left gluteus with increased FDG uptake. A pathological biopsy of the left gluteal mass (approximately 10x6 cm, as shown in **Figure 1A**) indicated invasive/metastatic adenocarcinoma. Further examination via colonoscopy revealed a neoplasm near the dentate line in the rectum, which was biopsied. The pathological results of the left gluteal mass and the colonoscopic biopsy were highly consistent, both indicating poorly differentiated adenocarcinoma. Immunohistochemical results showed positive for Villin, HER2 (1+), and Ki67 (80%+), while MLH1, PMS2, MSH2, and MSH6 were all positive. Genetic testing revealed a KRAS gene exon mutation, while NRAS, BRAF, and PIK3CA mutations were not detected, with the tumor being microsatellite stable (MSS). An MRI of the abdomen and pelvis further assessed organ metastasis, identifying metastatic lesions in the pelvis, multiple lymph nodes in the groin, and infiltrative lesions in the left gluteal skeletal muscle. The tumor was staged as T4bN2M1b IVB.

**Figure 2:**
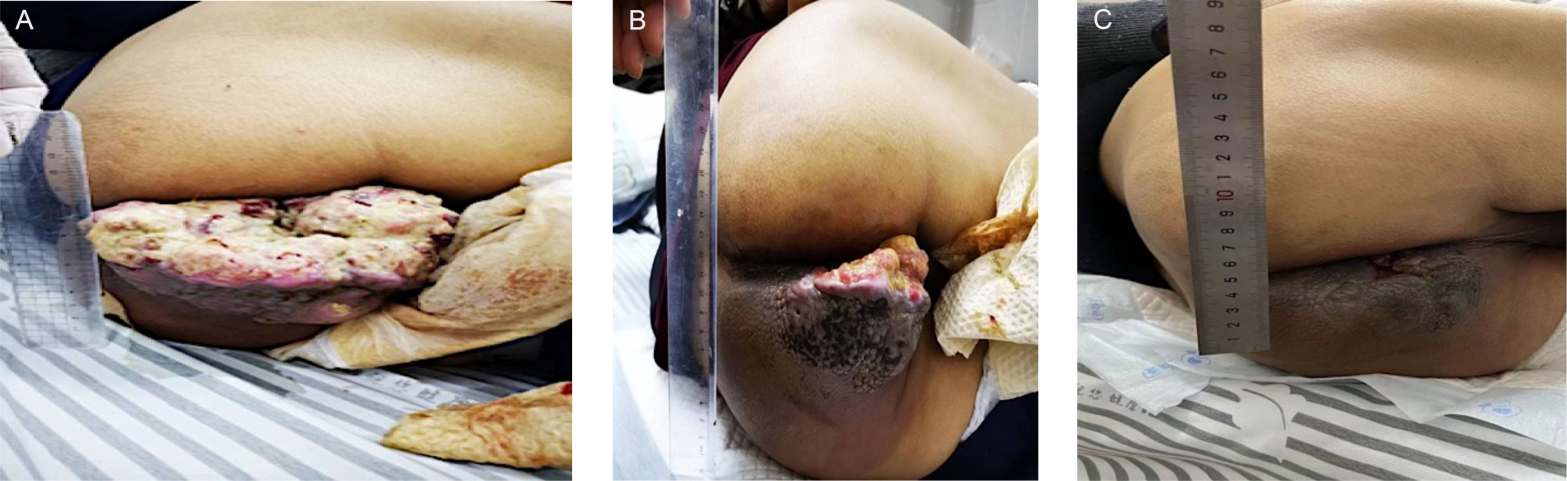
Treatment response of metastatic lesions in the patient’s buttock **(A)** with the large ulcerated lesion at initial diagnosis, **(B)** following one cycle of arterial embolization chemotherapy combined with two cycles of the mFOLFOX6 regimen, and **(C)** the outcome after four cycles of mFOLFOX6 chemotherapy.

The patient’s general condition was poor, with a height of 170.0 cm, weight of 44.0 kg, and ECOG performance status of 1. Considering the patient’s tumor burden and quality of life, and respecting the wishes of the patient and his family, a venous access port was established for supportive therapy. On October 20, 2023, the patient received a reduced-dose CAPEOX chemotherapy regimen (Oxaliplatin 130 mg/m² via IV; Capecitabine 1000 mg/m² orally, twice daily for two weeks, followed by a one-week break). After one cycle of treatment, the patient developed Grade IV bone marrow suppression and Grade III gastrointestinal reactions. There was no significant reduction in the gluteal mass. Following a multidisciplinary consultation, a percutaneous iliac artery angiography (as shown in **Figure 1B**) and tumor embolization chemotherapy were conducted in November 2023 (the treatment plan included 20 mg of lipiodol emulsion-doxorubicin), along with the implantation of an arterial chemotherapy pump (the arterial chemotherapy regimen was mFOLFOX6 plus Bevacizumab: Oxaliplatin 85 mg/m² over 2 hours, Leucovorin 400 mg/m² over 2 hours, 5-FU 400 mg/m² over 15 minutes, 5-FU 2400 mg/m² over 46 hours, and Bevacizumab 5 mg/kg over 1 to 1.5 hours, where m² represents body surface area). The patient tolerated the procedure well. After successfully completing two cycles of arterial infusion chemotherapy, the left gluteal mass significantly reduced (as shown in **Figures 2A, B**); After completing a total of four cycles of arterial infusion chemotherapy, and combining the patient’s hematologic parameters, such as CEA levels, with imaging assessments of the pelvis and upper chest, the patient’s left gluteal lesion was evaluated as having a partial response (PR) according to the RECIST v1.1 criteria. The maximum diameter of the lesion was reduced by approximately 90% (as shown in **Figure 2C**). During the treatment, the patient did not experience significant bone marrow suppression or liver and kidney function impairment, showing only mild fatigue. However, due to a severe pulmonary infection leading to respiratory failure, the patient ultimately passed away in February 2024, with a total progression-free survival (PFS) of 3 months.

## Discussion

The pathways of colorectal cancer metastasis include lymphatic spread, hematogenous distribution, and direct invasion (3). Interestingly, despite skeletal muscle constituting nearly 50% of total body mass and being well-vascularized, the incidence of rectal cancer with infiltration metastasis to the gluteal muscles is extremely low (8, 9). Asymptomatic intramuscular metastases may present inconspicuously (as indicated by increased FDG uptake in some studies) (10), with most patients initially presenting due to progressive pain and swelling (see **Table 1**). The pathophysiological mechanisms, optimal treatment modalities, and prognosis of intramuscular metastasis require further investigation.

**Table 1:** Overview of clinical characteristics in colorectal carcinoma patients with gluteal muscle metastases documented in the literature.

No.	Study	Age (Years)	Sex	Initial symptoms	Primary carcinoma	Site of muscle metastasis	Treatment regimens	Time interval (months)	Outcomes
1	Caskey CI (1988) (7)	62	Male	Lump and swelling	Transverse colon	Left gluteus medius muscle	NA	6	NA
2	Lampenfeld ME (1990) (14)	75	Female	Lump	Rectum	Left gluteus maximus and medius	surgery	24	NA
3	Yoshikawa H (1999) (15)	54	Male	Pain	Sigmoid colon	Right gluteus muscle	surgery	24	Died
5	Tunio MA (2013) (16)	28	Male	Pain	Transverse colon	Right gluteus maximus, Rectus abdominis muscle	surgery; radiotherapy; FOLFOX4	11	Alive
4	Buemi A (2019) (11)	69	Female	Pain	Right colon	Left gluteus muscle	surgery	7	Alive
6	Benzalim M (2023) (6)	36	Male	Swelling and pain	Colon and rectum	Bilateral thigh and gluteal muscles	chemotherapy	12	Alive

Several studies have commented on the relatively low incidence of skeletal muscle metastasis (11). Proposed hypotheses include changes in blood flow within skeletal muscles, a low incidence of micro vascularization, damage to cancer cells in skeletal muscle, and the production of low molecular weight non-protein factors that may inhibit tumor cell proliferation (12, 13). However, once intramuscular metastasis occurs, it often indicates a poor prognosis due to the potential for systemic spread (5). Currently, the main treatment approaches for patients with skeletal muscle metastasis include aggressive surgical resection, localized radiotherapy, and systemic chemotherapy (6, 7, 11, 14–16).

The patient described in this study presented with a progressively enlarging and ulcerated gluteal mass, signifying a late stage of diagnosis. In addition to muscular metastasis, there were already multiple metastatic lesions in the pelvic region and lymph nodes. The patient’s general condition was poor, with cachexia, placing him in a terminal state. In principle, palliative supportive care should have been the primary approach. However, the patient and his family expressed a strong desire for treatment. After thorough consultations involving specialists from interventional radiology, gastrointestinal surgery, nutrition, intensive care, and medical oncology, a comprehensive nutritional support plan was established.

A comprehensive review of six reported cases of colorectal cancer with gluteal muscle metastasis was conducted, with the associated treatment regimens and clinical outcomes detailed in **Table 1**. Among the identified patients, four received surgical intervention, two were treated with chemotherapy alone, and one patient underwent radiotherapy in conjunction with surgery. Additionally, we noted an early case from 1988 for which specific treatment details were not documented.

In formulating a personalized treatment plan for this patient, we undertook a thorough analysis delineating the distinctions between conventional and innovative therapeutic modalities. Traditional surgical resection was deemed unfeasible due to the extensive nature of the tumor, as complete excision could result in a large wound that would be difficult to heal, particularly in the context of the patient’s poor overall health and cachexia. Palliative radiotherapy was not pursued due to its requirements for the patient to maintain a prone position for extended periods, which could exacerbate pain and discomfort from the ulcerated mass. Additionally, the risk of extensive necrosis and potential bleeding from radiation treatment could further compound the patient’s condition. Although systemic second-line chemotherapy might have been considered, the patient’s poor general condition and malnutrition significantly increased the likelihood of suboptimal responses due to potential drug resistance. Locoregional transarterial chemotherapy is widely used in the treatment of liver cancer, characterized by high regional efficacy and minimal systemic adverse reactions. Interventional chemotherapy is effective for various gastrointestinal tumors, not just liver cancer (17, 18). Research shows that it works well for both conversion and palliative treatment in advanced gastric cancer with fewer side effects than systemic chemotherapy (19). Additionally, combining hepatic arterial infusion chemotherapy with systemic chemotherapy boosts response rates and survival in advanced pancreatic cancer (20). In recent years, there has been a gradual increase in reports regarding the use of locoregional chemotherapy for initially inoperable colorectal cancer (including both non-metastatic and metastatic CRC) (21). Therefore, intra-arterial chemotherapy was selected to deliver a concentrated dose of chemotherapy directly to the tumor area, aiming to alleviate the patient’s pain while enhancing overall quality of life through minimized systemic exposure and maximized localized treatment efficacy.

In a bold attempt to try an innovative approach, we implemented arterial embolization chemotherapy, an important modality in tumor interventional treatment. The combination of arterial embolization and chemotherapy is being reported for the first time in this context. This treatment paradigm was chosen based on the unique clinical circumstances of a patient with a significant ulcerative lesion due to sacral muscle metastasis, along with the presence of cachexia. This approach serves as a viable alternative, effectively balancing the need for local tumor control while minimizing systemic toxicity. This technique allows for the selective catheterization of tumor-supplying blood vessels via percutaneous puncture for administering chemotherapy drugs directly into the tumor. It can also involve subcutaneous drug pump administration and catheter-directed chemotherapy along with embolization, which has become essential for maximizing the anticancer efficacy of localized chemotherapy (22–24). The enhanced local drug delivery achieved through intra-arterial administration allows for higher concentrations of chemotherapy agents directly at the metastatic site, potentially improving efficacy while minimizing systemic exposure and associated side effects. Additionally, skeletal muscle has a rich vascular supply, which may facilitate improved drug uptake and retention compared to other metastatic sites with different vascular characteristics. Furthermore, the unique metabolic environment and lower interstitial pressure in skeletal muscles can enhance drug penetration and distribution.

This case demonstrates the potential efficacy of combined iliac artery embolization and intra-arterial chemotherapy for gluteal muscle metastases from colorectal cancer, as evidenced by the remarkable 90% tumor reduction shown in the follow-up imaging. However, the interpretation of these promising results must be tempered by the primary limitation of abbreviated follow-up due to the patient’s early demise at three months, which precluded assessment of the treatment’s durability and long-term outcomes. Despite this constraint, the significant local tumor response and improved quality of life observed in this terminal-stage patient suggest this approach warrants further investigation in similar challenging clinical scenarios, particularly for symptomatic palliation of rare metastatic patterns in colorectal cancer. Future prospective studies with standardized follow-up protocols would help validate these preliminary findings.

## Data Availability

The original contributions presented in the study are included in the article/supplementary material. Further inquiries can be directed to the corresponding author.
